# Treatment Modality and Quality Benchmarks of Aneurysmal Subarachnoid Hemorrhage at a Comprehensive Stroke Center

**DOI:** 10.3389/fneur.2018.00152

**Published:** 2018-03-15

**Authors:** Wengui Yu, Tapan Kavi, Tamara Majic, Kimberly Alva, Asma Moheet, Patrick Lyden, Wouter Schievink, Gregory Lekovic, Michael Alexander

**Affiliations:** ^1^Department of Neurology, Cedars-Sinai Medical Center, Los Angeles, CA, United States; ^2^Department of Neurosurgery, Cedars-Sinai Medical Center, Los Angeles, CA, United States; ^3^Department of Neurology, University of California Irvine Medical Center, Orange, CA, United States; ^4^Department of Neurology and Neurosurgery, Cooper University Hospital, Camden, NJ, United States; ^5^Division of Neurosurgery, House Clinic, Los Angeles, CA, United States

**Keywords:** aneurysm, subarachnoid hemorrhage, coiling, clipping, door-to-treatment time, outcome, length of stay, mortality rate

## Abstract

**Background:**

Aneurysmal subarachnoid hemorrhage (aSAH) is the most severe type of stroke. In 2012, the Joint Commission, in collaboration with the American Heart Association/American Stroke Association (AHA/ASA), launched the Advanced Certification for Comprehensive Stroke Centers (CSCs). This new level of certification was designed to promote higher standard of care for patients with complex stroke.

**Objective:**

The goal of this study was to examine the treatment modality and quality benchmarks of aSAH at one of the first five certified CSCs in the United States.

**Methods:**

Consecutive patients with aSAH at Cedars-Sinai Medical Center between April 1, 2012 and May 30, 2014 were included for this retrospective study. The ruptured aneurysm was treated with coiling or clipping within 24 h. All patients were managed per AHA guidelines. Discharge outcomes were assessed using modified Rankin Scale (mRS). The rate of aneurysm treatment, door-to-treatment time, rate of posttreatment rebleed, hospital length of stay (LOS), discharge outcome, and mortality rates were evaluated as quality indicators.

**Results:**

The median age (interquartile range) of the 118 patients with aSAH was 55 (19). Among them, 84 (71.2%) were females, 94 (79.7%) were transfers from outside hospitals, and 74 (62.7%) had Hunt and Hess grades 1–3. Sixty patients (50.8%) were treated with coiling, 52 (44.1%) with clipping, and 6 (5.1%) untreated due to ictal cardiac arrest or severe comorbidities. The rate of aneurysm treatment was 95% (112/118) with median door-to-treatment time at 12.5 (8.5) h and 0.9% (1/112) posttreatment rebleed. The median ICU and hospital LOS were 12.5 (7) and 17.0 (14.5) days, respectively. Coiling was associated with significantly shorter LOS than clipping. There were 59 patients (50%) with favorable outcome and 19 deaths (16.1%) at hospital discharge. There was no significant difference in discharge outcome between coiling and clipping.

**Conclusion:**

Care of aSAH at one of the early CSCs in the United States was associated with high rate of aneurysm treatment, fast door-to-treatment time, low posttreatment rebleed, excellent outcome, and low mortality rate. Coiling was associated with significant shorter LOS than clipping. There was no significant difference in discharge outcomes between treatment modalities.

## Introduction

Aneurysmal subarachnoid hemorrhage (aSAH) is the most devastating type of stroke (1). Ruptured cerebral aneurysms can be treated by surgical clipping or endovascular coiling. In 2002, the International Subarachnoid Aneurysm Trial (ISAT) showed that, in patients with ruptured intracranial aneurysms, suitable for both treatments, endovascular coiling is more likely to result in independent living at 1 year than surgical clipping (2). The benefit continues for at least 7 years (3). This landmark study has caused a paradigm shift for the treatment of cerebral aneurysms around the world (4, 5). Endovascular coiling has since become the mainstay in many medical centers (4–7). Due to technological advances and increasing fellowship opportunities for endovascular training, the use of coiling has also spread from large academic medical centers to small community hospitals across geographic regions (5).

Along the timeline, two retrospective studies demonstrated lower mortality rate after SAH at high-volume hospitals than that at low-volume hospitals in California and 18 States, respectively (8, 9). The mortality rate at the centers treating more than 35 cases a year was statistically and significantly lower than the centers treating less than 10 cases a year (27 vs 39%; odds ratio, 1.4) (9). However, such significant findings have not caused a paradigm shift in patient care. Although the overall mortality rate of aSAH has decreased over time (10, 11), most aSAH patients were still treated at low-volume centers in the United States (12). In the recent analysis of 31,973 patients with SAH from 685 hospitals participating in *Get With The Guidelines-*Stroke Registry, the median annual case volume was 8.5 (6.7–12.9) patients per hospital (12). The in-hospital mortality rate was 22.1% in the high-volume hospitals (12.9–94.5 cases per year) as compared to 29.5% in the low-volume hospitals (4–6.6 cases per year).

In 2012, The Joint Commission (TJC) launched Advanced Certification for Comprehensive Stroke Centers (CSCs) to improve the care of patients with complex stroke (13). TJC adopted a threshold of minimum of 20 annual cases of SAH among several requirements for CSC certification (14). Certified CSCs are required to perform 15 or more endovascular coiling or surgical clipping procedures for aneurysm, and to administer IV tPA to an average of 25 or more eligible patients.

However, in addition to mortality rate, there were no well-defined quality indicators for aSAH in the literature. The Agency for Healthcare Research and Quality patient safety indicators and the Centers for Medicare and Medicaid Services hospital-acquired conditions (HACs) are general metrics used to gauge the safety and quality of health care provided by health-care institutions. They were developed mainly to monitor adversary events and HACs. Given the significant risk of rebleed prior to aneurysm treatment, substantial cost of the care from hospitalization, and lack of reporting standards for aSAH (15, 16), we considered the rate of aneurysm treatment, door-to-treatment time, rate of treatment within 24 h, rate of posttreatment rebleed, hospital length of stay (LOS), outcome, and mortality rates as quality indicators.

Cedars-Sinai Medical Center was one of the first five medical centers in the country to have achieved CSC certification in 2012. In this study, we sought to examine the treatment modality and quality benchmarks of aSAH at a CSC.

## Patients and Methods

The daily handoff information on all patients admitted to the Neuroscience Intensive Care Unit (Neuro ICU) at Cedars-Sinai Medical Center was compiled as a skeletal database prospectively for quality improvement. Consecutive patients with aSAH between April 1, 2012 and May 30, 2014 were retrospectively screened and analyzed for aneurysm treatment and quality benchmarks. Patients with SAH secondary to trauma, arteriovenous malformation, or arteriovenous fistula were excluded from the study.

All patients were managed by the neurointensivist-led multidisciplinary team at the dedicated Neuro ICU per AHA/ASA and Neurocritical Care Society guidelines (15, 16). All patients with spontaneous SAH underwent CT angiography and/or digital subtraction angiography (DSA) for definitive diagnosis of cerebral aneurysms. Angiogram-negative SAH patients were further evaluated with repeat angiogram at day 7.

Surgical clipping or endovascular coiling of the ruptured aneurysm was at the discretion of an interdisciplinary team of two cerebrovascular surgeons, two neurointerventional radiologists, and a dual trained neurosurgeon. All ruptured aneurysms were treated within 24 h of hospital arrival. External ventricular drain (EVD) was placed by neurosurgery service for the patients with symptomatic hydrocephalus or diffuse SAH and intraventricular hemorrhage (IVH). Nimodipine was ordered for every patient for 21 days by default using standard admission orderset developed for the electronic medical record (EPIC Systems, Verona, WI, USA). It could only be held or the dose be reduced in case of hypotension or severe arrhythmia. Hypertonic saline and fludrocortisone were administrated for patients with hyponatremia from cerebral salt wasting. Routine surveillance for vasospasm included hourly neurological monitoring and daily transcranial Doppler measurements for 10–14 days. Symptomatic vasospasm was treated with normal saline bolus and pressor induced-hypertension (HTN). Intra-arterial vasodilators or balloon angioplasty was the treatment option for severe or refractory vasospasm per surgeon’s discretion. We adopted rapid EVD weaning protocol for appropriate patients (17). Ventriculoperitoneal (VP) shunt was placed for the patients who failed EVD weaning at least once.

Functional outcomes at hospital discharge were assessed using modified Rankin scale (mRS), which was abstracted from hospital discharge summary and progress notes by the Neuro ICU team or Neurosurgery service. Hunt and Hess grade was abstracted directly from the chart review. Fisher grade and mRS were independently assessed by three experienced neurologists (WY, TM, and TK). Any disagreement was adjudicated per consensus. Favorable functional outcome was defined as mRS ≤3 while poor functional outcome was defined as mRS ≥4. The study was approved by the Institutional Research Board.

Normally distributed data were reported as mean ± SD, and non-parametric data were reported as median with interquartile range (IQR). Statistical analysis was performed using Fisher’s exact test and the unpaired student’s *t*-test for binary and continuous variables, respectively. *p-*Value <0.05 was considered statistically significant in subgroup analysis.

## Results

**Figure 1:**
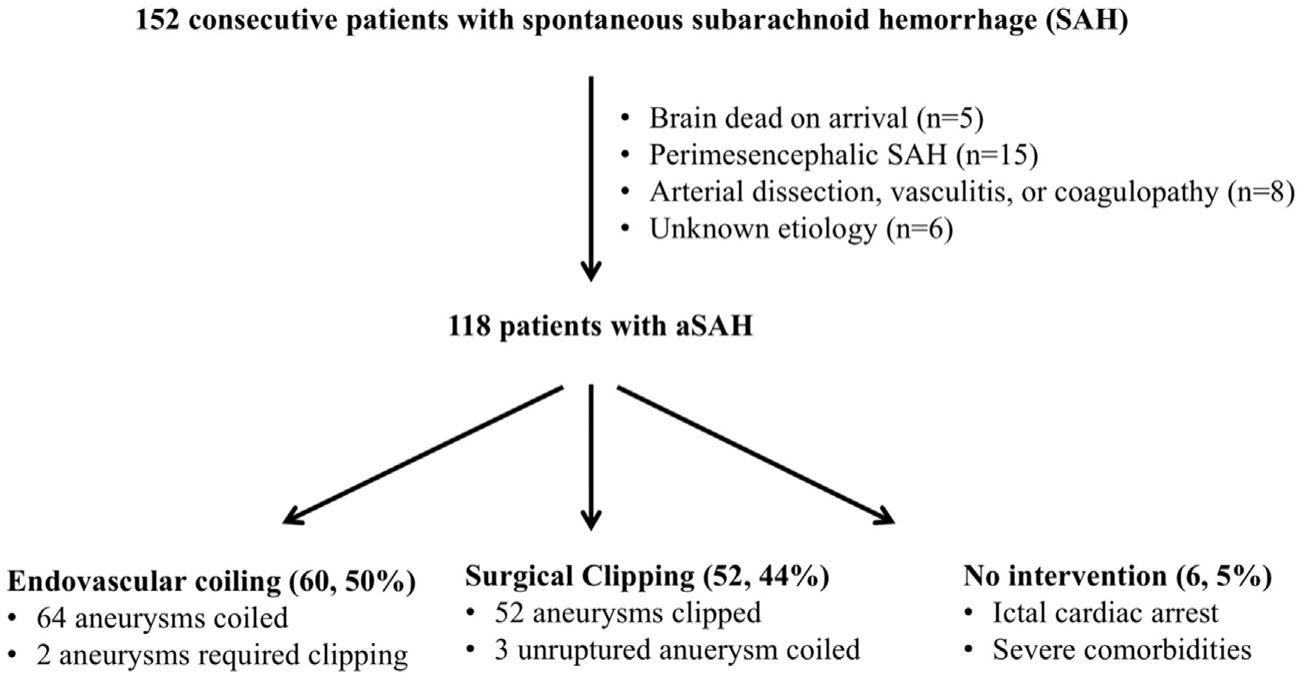
Screening of patients with aneurysmal subarachnoid hemorrhage (aSAH).

One hundred and fifty-two patients were found to have spontaneous SAH during the study period (Figure 1). Thirty-four patients were excluded from the study due to brain death on arrival without vessel image (*n* = 5), perimesencephalic SAH (*n* = 15), arterial dissection, vasculitis or coagulopathy (*n* = 8), or unknown etiology (*n* = 6). One hundred and eighteen patients were confirmed to have aSAH by CTA and/or DSA.

The demographics and clinical characteristics of the patients with aSAH were shown in Table 1.

**Table 1:** Demographics and clinical characteristics of patients with aneurysmal subarachnoid hemorrhage.

Variables	Numbers	%
No. of patients	118	
Age [interquartile range (IQR)]	55 (19)	
Female sex	84	71.2
History of HTN	63	53.4
Direct emergency department (ED) admission	24	20.3
Transfer from outside hospital	94	79.7
Initial GCS (IQR)	14 (7)	
Hunt and Hess grade		
1	5	4.2
2	50	42.4
3	19	16.1
4	18	15.3
5	26	22.0
Fisher grade		
1	7	5.9
2	16	13.6
3	21	17.8
4	74	62.7
Aneurysm location		
Anterior circulation	98	83.0
Posterior circulation	20	17.0
Aneurysm treatment		
Endovascular coiling	60	50.8
Surgical clipping	52	44.1
None	6	5.1
Rate of aneurysm treatment	112/118	95
Door-to-treatment time (h) (IQR)	12.5 (8.5)	
Direct ED admission	11.8 (10.2)	
Transfer from outside hospitals	12.4 (4.5)	
Rate of treatment within 24 h	112/112	100
Rate of posttreatment rebleed	1/112	0.9
External ventricular drain placement	69	58.5
Ventriculoperitoneal shunt	15	12.7
ICU length of stay (LOS) (days) (IQR)	12.5 (7)	
Hospital LOS (days) (IQR)	17.0 (14.5)	
mRS at discharge		
0–3	59	50
4–5	40	33.9
6	19	16.1

The median age with IQR was 55 (19). Eighty-four patients (71.2%) were females. Sixty-three patients (53.4%) had a history of HTN. Twenty-four patients (20%) were direct admissions from the emergency department (ED) and 94 patients (80%) were transferred from community hospitals. The median GCS score was 14 (7). Of the 118 patients, 74 (62.7%) were Hunt and Hess grades 1–3 and 44 (37.3%) were Hunt and Hess grades 4–5. Ninety-eight patients (83%) had aneurysms in the anterior circulation and 20 patients (17%) had posterior circulation aneurysms. Sixty patients (50.8%) were treated with endovascular coiling and 52 (44.1%) were treated with surgical clipping. Six patients (5.1%) were not treated due to ictal cardiac arrest or severe comorbidities. The rate of aneurysm treatment was 95% (112/118).

The median door-to-treatment time with IQR was 12.5 (8.5) hours. The rate of treatment within 24 h was 100%. There was no significant difference in treatment time between ED admissions and transfers from outside hospitals (*p* = 0.52). One patient rebled and died 6 days after endovascular coiling of a small ICA aneurysm. The rate of posttreatment rebleed was 0.9% (1/112).

Sixty-nine patients (58.5%) had EVD placement for hydrocephalus, severe IVH, or evidence of elevated intracranial pressure. Fifteen patients (12.7%) did not tolerate EVD weaning and required VP shunt placement.

The median ICU and hospital LOS with IQR were 12.5 (7) and 17.0 (14.5) days, respectively. Fifty-nine patients (50%) had favorable functional outcome with mRS 0–3 at hospital discharge. Forty-one patients (34.7%) had severe disability and were discharged to acute rehabilitation or skilled nursing facilities. The mortality rate of the cohort was 16.1% (19/118). Seven patients (5.9%) died from ictal cardiac arrest or brain-stem herniation from initial hemorrhage, 4 (3.4%) from pretreatment rebleed in outside hospitals or during transfer, 1 (0.9%) from posttreatment rebleed, and 7 (5.9%) from poor grade SAH and withdrawal of life support per patient/family wishes. The mortality rate of poor grade SAH was 30.5%.

As shown in Table 2, slightly more patients were treated with endovascular coiling than with surgical clipping (60 vs 52). There was no significant difference in age, sex, history of HTN, initial GCS, Hunt and Hess grade, and Fisher grade between the coiling and clipping groups. Of the 92 patients with anterior circulation aneurysms, 42 (43.5%) were treated with endovascular coiling and 50 (54.3%) with surgical clipping. In contrast, of the 20 patients with posterior circulation aneurysms, 18 (90%) underwent endovascular coiling and only two were treated with surgical clipping (*p* < 0.005). The coiling group had statistically and significantly shorter LOS in the ICU than the coiling group (*p* < 0.05). There was no significant difference in door-to-treatment time, EVD placement, VP shunt requirement, hospital LOS, and discharge outcome between the two groups. The outcome of the coiling group was non-significantly worse than the clipping group, likely due to older age, higher Hunt and Hess grade, and higher proportion of patients with posterior circulation aneurysms in the coiling group.

**Table 2:** Characteristics and quality benchmarks between the coiling and clipping groups.

Variables	Coiling	Clipping	*p*-Value
No. of patients	60	52	
Mean age [interquartile range (IQR)]	57.0 (26.3)	54.5 (17.5)	0.44
Female sex	42 (70%)	39 (75%)	0.88
History of HTN	34 (56.7%)	29 (55.7%)	1.0
Initial GCS (IQR)	14.0 (7.3)	14.0 (5.3)	0.15
Hunt and Hess grade (IQR)	3.0 (2.0)	2.2 (2.0)	0.29
Grades 1–3	38 (63.3%)	38 (73.1%)	0.63
Grades 4–5	22 (36.7%)	14 (26.9%)	0.43
Fisher grade (IQR)	4.0 (1.0)	4.0 (1.0)	0.97
Grades 0–3	24 (40.0%)	21 (40.4%)	0.97
Grades 4	36 (60.0%)	31 (59.6%)	0.98
Aneurysm location			
Anterior circulation	42 (43.5%)	50 (54.3%)	0.32
Posterior circulation	18 (90%)	2 (10%)	<0.005
Door-to-treatment time (h) (IQR)	11.1 (8.1)	13.4 (9.0)	0.72
External ventricular drain placement	35 (58.3%)	31 (59.6%)	1.0
Ventriculoperitoneal shunt	8 (13.3%)	8 (15.4%)	0.8
ICU length of stay (LOS) (days) (IQR)	11.0 (7.3)	13.0 (6.0)	<0.05
Hospital LOS (days) (IQR)	15.5 (12.3)	18.5 (11.5)	0.075
mRS at discharge (IQR)	4.0 (3.0)	3.0 (2.0)	0.43
0–2	19 (31.7%)	21 (40.4%)	0.58
3–4	22 (36.6%)	21 (40.4%)	1.0
5–6	19 (31.7%)	10 (19.2%)	0.34

In patients with anterior circulation aneurysms, there were also no significant differences in demographics, clinical characteristics, and outcome between the coiling and clipping groups (Table 3). However, the coiling group had statistically significant shorter LOS in the ICU (*p* < 0.05) and in the hospital (*p* < 0.05) than the clipping group.

**Table 3:** Coiling vs clipping for anterior circulation aneurysms.

Variables	Coiling group	Clipping group	*p-*Value
No. of patients	42	50	
Mean age [interquartile range (IQR)]	54.0 (21.8)	55.5 (18.3)	0.83
Female sex	31 (74%)	37 (74%)	1.0
Initial GCS (IQR)	15.0 (7.8)	14.0 (4.8)	0.17
Hunt and Hess grade	2.5 (2.0)	2.0 (1.8)	0.38
Fisher grade	4.0 (1.0)	4.0 (1.0)	0.87
ICU length of stay (LOS) (days) (IQR)	11.0 (6.0)	13.0 (4.8)	<0.05
Hospital LOS (days) (IQR)	14.5 (10.8)	18.5 (10.8)	<0.05
mRS at discharge (IQR)	3.0 (2.8)	3.0 (2.0)	0.99
0–2	16 (38%)	20 (40%)	1.0
3–4	15 (36%)	20 (40%)	0.84
5–6	11 (26%)	10 (20%)	0.63

## Discussion

We report the treatment modality and quality benchmarks of aSAH at one of the first five CSCs in the United States. As a high-volume CSC, we treated 118 aSAH between April 1, 2012 and May 30, 2014. Among them, 60 were treated with coiling and 50 with clipping. Most patients with posterior circulation aneurysms (18/20) were treated with coiling. The rate of aneurysm treatment was 95% (112/118) with median door-to-treatment time at 12.5 (8.5) h and 0.9% (1/112) posttreatment rebleed. Fifty-nine patients (50%) had favorable functional outcomes (mRS 0–3) at hospital discharge. The mortality rate was 16.1%.

Our treatment strategy of ruptured aneurysms reflects the recent paradigm shift and standard care per AHA/ASA guidelines (2–4, 16). Although coiling is associated with better functional outcome (2, 3), clipping remains an important treatment option for younger patients and patients with small, wider neck aneurysms, or intracerebral hematoma (6, 7). Our clipping rate appeared to be higher than most centers likely due to strong surgical expertise. Consistent with results from other tertiary neurosurgical centers (6, 7), there was no significant difference in discharge outcome between coiling and clipping. Our data did confirm the previous report on longer LOS from clipping than coiling (18).

Most of our aSAH patients (80%) were transfers from outside hospitals, partly because we were the first certified CSC in the Los Angeles and the AHA/ASA recommendation on early transfer of patients with aSAH from low-volume facilities to high-volume tertiary centers (15).

The mortality rate at our CSC was 16.1% as compared to 22.1% at the high-volume centers (12.9–94.5 case volume/year) from the *Get with the Guidelines*-Stroke Registry (12). The lower mortality rate may be due to physician expertise in both clipping and coiling, high rate and early treatment of ruptured aneurysms, a dedicated Neuro ICU, neurointensivist-led multidisciplinary team, and prompt management of medical or surgical complications (19–22).

The treatment of aSAH at high-volume centers was shown to be associated with lower rates of in-hospital mortality and better odds of good functional outcome after adjusting for variables (23–26). In a study of 25 specialty centers in England, the annual institutional caseload of SAH was found to be inversely related to 6-month mortality (23). Each 100-patient increase in institutional volume was associated with a 24% relative reduction in 6-month mortality after SAH.

The mortality rate was much lower at 11.3% in a recent large multicenter study of aSAH treated at regional tertiary neurosurgical centers in the UK and Ireland (27), Other quality benchmarks, including the rates of preoperative rebleed (4.9%), aneurysm treatment (93.5%), median time to treatment [1(2) days], LOS [15(14)], and good recovery at discharge (54.6%), were all comparable to that of our single center study. Higher coiling rate (75%) and less transfer of poor grade SAH to tertiary centers in the UK due to clinical futility concerns might be some of the factors contributing to the lower rate of mortality (27).

Of note, the mortality rate should not be overly weighted as an outcome benchmark for performance improvement. For patients with poor grade SAH and terminal illness or multiorgan failure, the quality of life and patient’s wishes are relevant as well. In our cohort, seven patients (5.9%) died due to withdrawal of life support per patient wishes or family decision to prevent futile care and prolonged suffering. Case fatality was 11.8% (3.8–19.9%) lower in Japan than it was in Europe, the USA, Australia, and New Zealand (1, 28). However, the hospital LOS in Japan was more than 10 days longer, suggestive of prolonged life support for poor grade patients with terminal illness (28).

Our data can potentially be used as quality benchmarks for the readers or other hospitals to assess and compare for quality improvement and clinical studies. The strength of our study is that the data quality is much better than the administrative data from the National Inpatient Sample (NIS) and *Get with the Guidelines*-Stroke Registry (12, 23–26). Our skeletal database was compiled prospectively with 100% capture of SAH cases. The charts were reviewed and abstracted by experienced neurointensivists. All consecutive patients with aSAH were included for the analysis. In contrast, the administrative data from the NIS and *Get with the Guidelines*-Stroke Registry contain limited clinical details and inaccurate identification of aSAH. For example, of the 32,336 discharges from NIS between 2002 and 2010, only 13,398 patients (41.4%) were treated with clipping or coiling (25).

The major limitation of our study is that it is retrospective with only outcome data at hospital discharge. Other limitations include: (a) relatively small sample size from a single center that is not powered to detect outcome difference between clipping and coiling; (b) the empirical use of intra-arterial vasodilators or balloon angioplasty for the treatment of severe or refractory vasospasm without accepted standard; (c) data on other potential quality indicators, such as the rate of treatment with nimodipine, delayed cerebral injury, CSF infections or discharged destination, were not collected; and (d) not clear if the CSC certification improved the care of aSAH. It would be helpful to have data before the CSC certification for comparison.

## Conclusion

We report treatment modality and quality benchmarks of aSAH at one of the first five certified CSCs in the United States. Management of aSAH at CSC was associated with high rate of aneurysm treatment, fast door-to-treatment time, low rate of posttreatment rebleed, excellent outcome and low mortality rate at hospital discharge. There was no significant difference in functional outcome between coiling and clipping groups. However, coiling was associated with significant shorter LOS than clipping.

## Ethics Statement

This study was retrospective and the protocol was approved by the Institutional Research Board at Cedars Sinai Medical Center.

## Author Contributions

WY contributed to study design, data acquisition, analysis, interpretation, drafting, and finalizing the manuscript. TK, TM, and KA contributed to acquisition of data and analysis. AM, PL, WS, GL, and MA contributed to data interpretation and critical revision of the manuscript.

## Conflict of Interest Statement

WY has received compensation for activities with Stryker as a consultant. MA has received compensation for activities with Penumbra Inc., as a consultant. Other co-authors have nothing to disclose.
